# Austenite Growth Behavior and Prediction Modeling of Ti Microalloyed Steel

**DOI:** 10.3390/ma17133236

**Published:** 2024-07-01

**Authors:** Jun Wang, Man Liu, Lifan Wang, Ping He, Haijiang Hu, Guang Xu

**Affiliations:** The State Key Laboratory of Refractories and Metallurgy, Key Laboratory for Ferrous Metallurgy and Resources Utilization of Ministry of Education, Wuhan University of Science and Technology, Wuhan 430081, China; junwang@wust.edu.cn (J.W.); wlf12012023@163.com (L.W.); w3176902049@163.com (P.H.); huhaijiang@wust.edu.cn (H.H.); xuguang@wust.edu.cn (G.X.)

**Keywords:** Ti microalloyed steel, austenite grain, precipitation, heating rate, model

## Abstract

Previous studies on the austenite grain growth were mostly based on a fixed temperature, and the relationship between the austenite grain and austenitizing parameters was fitted according to the results. However, there is a lack of quantitative research on the austenite grain growth during the heating process. In the present work, based on the diffusion principle of the controlled Ti microalloying element, the diffusion process of carbonitrides containing Ti during the heating process was analyzed. Combined with the precipitation model and the austenite growth model, the prediction model of austenite grain growth of Ti microalloyed steel during different heat treatment processes was established. The austenite grain size versus the temperature at four different heating rates of 0.5, 1, 10, 100 °C/s was calculated. The grain growth behavior of austenite during the heating process of Ti microalloyed steel was studied by optical microscope, scanning electron microscope and transmission electron microscope. The experimental data of the austenite grain size was in good agreement with the calculation by the proposed model, which provides a new idea for the prediction of austenite grain size in non-equilibrium state during the heating process. In addition, for Ti-containing microalloyed steels, the austenite grain size increased with the increasing heating temperature, while it changed little by further prolonging isothermal time after certain heating time, which was related to the equilibrium degree of the precipitation and the dissolution of Ti element. The austenite grain coarsening temperature of the tested Ti microalloyed steel was estimated within 1100~1200 °C.

## 1. Introduction

Austenite grain size is one of the main factors affecting the strength and toughness of steels because the grain size directly determines the phase transformation kinetics during the subsequent cooling process [1]. Different austenite grain sizes lead to significant discrepancies in the mechanical properties of the materials, especially in welding zones [2]. Therefore, it is very important to control the austenite grain size of hot-rolled strips. Scholars have proposed many methods to effectively control the austenite grain size [3]. From the viewpoint of thermal mechanical control processing, the austenite grain size can be refined by reducing the rolling temperature to inhibit recrystallization. On the other hand, microalloying elements of Nb, V, Ti are also used to refine austenite grain through the pinning effect of precipitations [4]. The pinning-force of precipitates on austenite grain boundaries depends on the size and volume fraction of precipitated particles [5]. Therefore, the coarsening or dissolution of microalloying carbonitrides directly affect the austenite grain size.

There are many publications about the effect of the precipitated particles on the austenite grain size [6]. According to the previous experimental procedures, the steels are usually heated from room temperature to the austenitizing temperature and held for a period of holding time (such as 30 min) followed by water quenching. Afterwards, the primary austenite grain boundaries are revealed by the specific corrosion method [7], and the austenite grain size is measured based on metallographic images. However, this method can only measure the static austenite grain size, and the growth behavior of austenite grains during the heating process cannot be clearly defined. Additionally, the effects of heating rate and non-isothermal process on the grain growth of the reversed austenite have also been studied. Militzer et al. [8] clarified the influence of the pinning force on the austenite grain growth by introducing a pinning effecting coefficient of precipitates. They stated that austenite grain size was a function of heating rate and heating temperature, and different heating temperatures corresponded to different critical austenite grain sizes. Andersen and Grong [9] proposed a mathematical relationship model between the austenite grain size and precipitation coarsening, which could be used to calculate the average austenite grain size at different austenitizing parameters. The coarsening of precipitated particles under continuous heating and cooling conditions were calculated by the classical Lifshitz-Slyovoz-Wagner (LSW) theoretical differential equation. Banerjee et al. [10] studied the effects of ultra-rapid heating (up to 1000 °C/s) rate and temperature on the growth kinetics of non-isothermal austenite grains. They found that the size distribution of precipitated particles also affected the austenite growth, and finally proposed an empirical model about the relationship between austenite grain growth kinetics and the solid solution of Nb carbonitrides. Although many above works tried to quantitatively describe the austenite grain growth during a non-isothermal process, most of them still used the equilibrium data at an austenitizing temperature without correction. There are few studies to predict the growth behavior of austenite grains in non-equilibrium state during heating process.

The traditional experimental method for establishing austenite grain growth model mainly includes the following two ways [11,12]. Through thermal simulation experiment and metallographic analysis, austenite grains are observed and the measured grain sizes are used to fit the austenite grain growth model. Another method is that the austenite grain growth process is observed by in-situ high-temperature microscope, and the austenite grain growth model is established based on Hillert model or Burke-Turnbull model [13]. The traditional austenite growth models are only applicable to the corresponding experimental steels. In the present work, the austenite grain growth rules of a low carbon microalloyed steel during different heating processes were analyzed, and the quantitative relationship between the volume fraction, the diameter of precipitates and the austenite grain size was analyzed. The prediction model of austenite grain growth of Ti-microalloyed steels during the heating process was established, which provides a theoretical guidance for the control of austenite grain size of low carbon Ti-microalloyed steels. Compared with the traditional austenite growth model and the non-equilibrium austenite growth model, the proposed model has a wider range of applications.

## 2. Modeling

### 2.1. Precipitation Model

Many studies have reported the solid solution behavior of Ti carbonitrides in austenite, and also given the solid solubility product formula of TiC and TiN in austenite [14,15,16]. The solubility products of TiC and TiN differ greatly in different literatures, some of which are derived theoretically while others are fitted with experimental data. The main reasons for the difference are the varied composition systems and lacking of the effect of other alloying elements on the solution of microalloying elements in most publications. Lei et al. [14] adopted thermodynamic phase diagram calculation method to deduce the solid solubility product model of the precipitation of microalloying elements in steels by considering influences of alloying elements into account. The specific expressions were as follows:

(1) $${log}^{\gamma }\left[Ti\right][C]=3.77-\frac{8339}{T}+\left(-0.002+\frac{14.2}{T}\right)\left[Mn\;wt.\%\right]+\left(-0.009+\frac{84.6}{T}\right)\left[Ni\;wt.\%\right]+\;\left(0.019+\frac{12.3}{T}\right)\left[Cr\;wt.\%\right]+\left(-0.017+\frac{52.1}{T}\right)\left[Mo\;wt.\%\right]$$

(2) $${log}^{\gamma }\left[Ti\right]\left[N\right]=4.37-\frac{15029}{T}+\left(0.009+\frac{36.5}{T}\right)\left[Mn\;wt.\%\right]\left(-0.014+\frac{73.4}{T}\right)\left[Ni\;wt.\%\right]+\;\left(0.004+\frac{122.9}{T}\right)\left[Cr\;wt.\%\right]+\left(-0.036+\frac{63.4}{T}\right)\left[Mo\;wt.\%\right]$$

where [Ti], [C] and [N] are the dissolved amounts (wt.%) of elements Ti, N and C in austenite, respectively; *T* is the absolute temperature (K). The solid solubility products of TiC and TiN of the experimental steel can be calculated by Formulas (1) and (2), respectively. In the present work, by substituting the composition of the experiment steel into Formulas (1) and (2), the solid solubility products of TiC and TiN are given in Formulas (3) and (4), respectively.

(3) $${log}^{\gamma }\left[Ti\right][C]=3.78-\frac{8307}{T}$$

(4) $${log}^{\gamma }\left[Ti\right][N]=4.39-\frac{14902}{T}$$

The volume fraction of precipitates (*f_v_*) is calculated by combining the composition and the temperature of the experimental steel. It needs to be pointed out that the result of *f_v_* calculated here is the equilibrium state. That means the dissolution rate of precipitates is the same as the crystallization rate, that is, the concentration of precipitates in the matrix is unchanged. Thus, the calculated *f_v_* is the minimum value at a fixed temperature (any temperature within the austenite region) during the reheating process. Meanwhile, the completed solution temperatures of TiC and TiN are calculated to be 1150 °C and 1450 °C, respectively. During the heating process, the precipitation and dissolution of the second phase particles do not reach the equilibrium state, and with the increase of the holding time, the volume fraction of precipitates decreases until the equilibrium state is reached. Therefore, we introduce the maximum diffusion distance of precipitated particles to correct the actual volume fraction. The maximum distance ($L_{\max}$) between precipitated particles at a heating temperature can be estimated as Equation (5).

(5) $$L_{\max}={\left(\int_{t_{0}}^{t}D_{Ti}dt\right)}^{1/2}$$

(6) $$D_{Ti}(m^{2}\cdot s^{-1})=2.8\times 10^{-4}exp(-286000/RT)$$

where $D_{Ti}$ is the diffusion coefficient of Ti element [17], and R is the gas constant (8.314 J•mol^−1^•K^−1^). The diffusion coefficient of Ti element changes with the temperature during the heating process, so the calculated result of Formula (5) is not only related to the time but also the temperature. During different heat treatment processes, the heating rate affects the duration time at different temperature segments, thus the heating rate affects the maximum distance ($L_{\max}$) of particles precipitated at the corresponding temperature. With the fixed time interval designed as the calculating node and the corresponding increased temperature, the maximum distances ($L_{\max}$) of precipitated particles at different heating rates are obtained by the cyclic calculation. Here, it is assumed that the pinning effects of TiC and TiN in austenite depend the particle diameter and the volume fraction. The pinning force of TiC and TiN particles with the same shape and size may be different due to different type. The effect of different morphology of particles such as the square and round shapes was ignored. The initial radius of precipitates is 2 nm based on following experimental results. The diffusion distance *L* is related to the volume fraction $f_{v}$ and the initial size $r_{0}$ of precipitated particles, which is specifically expressed as Equation (7) [10].

(7) $$f_{v}\times 4\pi L^{3}/3=4\pi {r_{0}}^{3}/3$$

When the diffusion interaction of precipitated particles is not considered, $L_{\max}$ = *L*, so Equation (8) can be obtained.

(8) $$f_{v}=\frac{r_{0}^{3}}{L_{max}^{3}}$$

Then, the volume fraction calculated by Formula (8) is compared with the minimum value of $f_{v-min}$ obtained from Equations (3) and (4). If the value of $f_{v}$ is larger than $f_{v-min}$, $f_{v}$ is taken for the subsequent calculation, otherwise $f_{v-min}$ is used.

Ostwald ripening is the aggregation and growth process of the second precipitates, which occurs after the precipitation is completed [18]. When the volume fraction of the second phase remains unchanged, the total interface area would decrease if the size of the second phase increases, resulting in a decrease in the interface energy of the system, which is the driving force of the Ostwald ripeness process [19]. The Ostwald ripening rates of different second phase systems are different, which leads to a significant discrepancy in the actual size of the second phase. The Ostwald ripening rates of different second phase systems are different due to the varied chemical composition, structure, reaction mechanism and external conditions. The changes of the particles in steels during the heating process conform to Ostwald ripening equation expressed as follows [20].

(9) $$r^{3}-{r_{0}}^{3}=K_{p}t$$

(10) $$K_{p}=\frac{8\gamma D_{Ti}V_{m}}{9RT}$$

where *r* is the size of precipitate, *t* is the time,$\;K_{p}$ is the ripening rate, *γ* is the interface energy of the precipitation, $V_{m}$ is the precipitation molar volume, $D_{Ti}$ is the diffusion coefficient of Ti element, R is the ideal gas constant and *T* is the absolute temperature.

### 2.2. Austenite Growth Model

The austenite grain size increases with increasing the austenitizing temperature. When the heating temperature is constant, the austenite grain size has a threshold value with the increase of holding time, that is, different austenitizing temperatures correspond to different maximum austenite grain sizes. The solid solubility of precipitates increases with increasing the isothermal temperature, but there is a saturation limit of solid solubility. When the saturation is reached, the redissolution of precipitates does not increase with extending time. When the driving force of austenite grain growth and the pinning force of precipitated particles reach a thermodynamic equilibrium state, the austenite grains reach the limiting size.

The growth rate of austenite grains can be expressed by Formula (11) [21].

(11) $$\frac{dD}{dt}=M(P_{D}-P_{Z})$$

where *D* is the austenite grain size, *M* is the mobility of the grain boundary, *P_D_* is the driving pressure for the grain growth, and *P_Z_* is the pinning pressure induced by precipitates. The grain boundary mobility, *M*, is given by:

(12) $$M=M_{0}exp(-\frac{Q}{RT})$$

where *M*_0_ is a pre-exponential factor, *Q* is an activation energy, R is the gas constant, and *T* is the absolute temperature. The driving pressure can be expressed as:

(13) $$P_{D}=\frac{4}{D}\gamma$$

where *γ* is the grain boundary energy. Zener derived a simple approach to estimate the pinning pressure [22].

(14) $$P_{Z}=\frac{3f_{v}}{2r}\gamma$$

where, *fv* is the volume fraction of precipitates and *r* is the mean particle radius. Zener-Smith proposed a mathematical relationship model of austenite grain growth related to the precipitation volume fraction and size [23]:

(15) $$D=4r/3f_{v}$$

In fact, not all the precipitated particles have the pinning effect on the austenite grain boundaries, only the precipitates distributed on the grain boundaries hinder the grain boundary migration. Therefore, scholars continuously revised the above model, and the overall form can be expressed as:

(16) $$D=k\frac{r}{f_{v}^{n}}$$

where *k* and *n* are constants, and the specific values are shown in Table 1. Nishizawa optimized *k* and *n* values by combining theoretical derivation and a large number of experimental results [24], and found that when *k* was 8/3 and *n* was 2/3, the calculated values were in good agreement with the experimental values. The steel tested in the present work are similar to Nishizawa’s, thus the selected austenite growth model is expressed as follows:

(17) $$D=8r/3f_{v}^{2/3}$$

## 3. Material and Method

**Figure 1:**
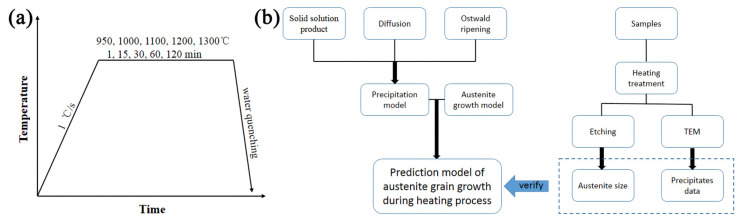
(**a**) The schematic diagram of heat treatments and (**b**) the flow chart of each design step.

The precipitation model and the austenite growth model were established in the second section. The austenite grain size during the heating process can be predicted by combining precipitation model and austenite growth model. In order to verify the proposed models, austenitizing experiments were conducted. The chemical composition of the experimental steel was Fe-0.075C-1.8Mn-0.5Cr-0.12Ti-0.0035N (mass fraction, %). The steel composition was measured by inductively coupled plasma mass spectrometer (America PerkinElmer NexION 1000G) and high frequency infrared ray carbon sulphur analyser (America CS744). The cast ingot was forged, and then cut into two small billets with the size of 180 mm (length) × 150 mm (width) × 70 mm (thickness). Firstly, the billet was isothermal holding at 1250 °C for 2 h. Secondly, the billet was subjected to the roughing rolling at 1130 °C and the finishing rolling at 950 °C, and the final thickness of the plate was 14 mm. Finally, the plate was water cooled to 550 °C followed by air cooling to room temperature. Metallographic microscope (OM, German Leica DM2700M) and scanning electron microscope (SEM, Oxford Nova 400 Nano) were used to detect the initial microstructure of the sample corroded by 4% nital solution. Cylindrical samples with diameter of 5 mm and height of 10 mm were machined for heat treatments shown in Figure 1a. Samples were heated to 950 °C, 1000 °C, 1100 °C, 1200 °C, 1300 °C, respectively, at 1 °C/s with holding times of 1, 15, 30, 60, 120 min, respectively. The heating process is carried out with the nitrogen protective gas. Then, twenty-five groups of samples were quenched with ice saline water to room temperature. The purpose of quenching is to ensure that the austenite grain size is unchanged during the cooling process, additionally, retain the original state of precipitates at individual austenization temperature. It is reported that the acicular austenite was easily formed when a hypoeutectoid steel was heated to the austenite transition temperature at a heating rate faster than 100 °C/s or slower than 0.5 °C/s [26]. Therefore, the heating rate of 1 °C/s was adopted in the present study for the formation of blocky austenite. The original austenite grains of all samples after heat treatments were observed after the specific corrosion method, as stated as follows. After grinding and polishing, the quenched samples were corroded in a water bath at 65 °C for 5 min with mixed liquid of 20 mL saturated bitric acid solution +10 mL sodium dodecyl benzene sulfonate aqueous solution and 2~4 drops of saturated HCl. The carbon replica extraction experiment was carried out to observe the number and size distribution of precipitated particles on a JEM-2100F transmission electron microscopy (TEM). The flow chart of design shown in Figure 1b.

## 4. Result

### 4.1. Original Microstructure

**Figure 2:**
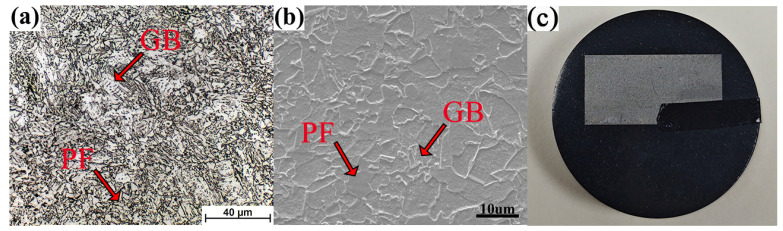
Original microstructure of the hot-rolled plate: (**a**) OM image; (**b**) SEM image; (**c**) sample image; PF-polygonal ferrite; GB-granular bainite.

Figure 2 gives the original microstructure and the sample of the hot-rolled plate. The initial microstructure consisted of polygonal ferrite (PF) and granular bainite (GB) with small size. Due to the addition of 0.12 Ti wt.%, the grain size of the original austenite was supposed to be small. Then the grain size of ferrite or bainite was further refined by the rapid water cooling and large supercooling degree of austenite. The average grain size of ferrite was measured to be 8.3 ± 2.8 μm by the area method based on as least 10 SEM images.

### 4.2. Austenite Grain after Austenization

**Figure 3:**
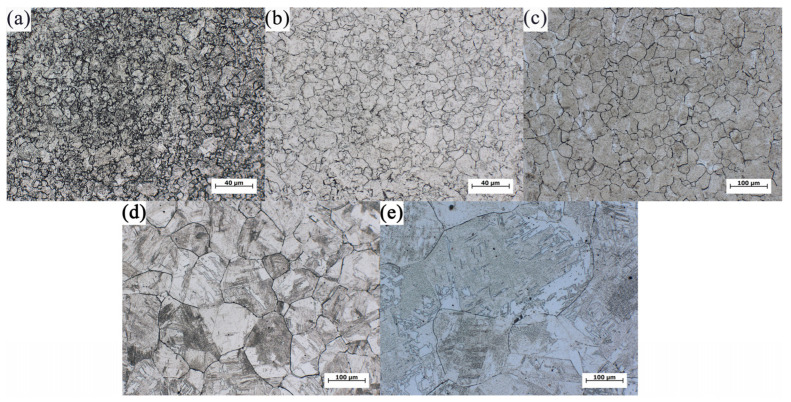
The austenite grain morphology of samples after holding at different austenitizing temperatures for 1 min: (**a**) 950 °C; (**b**) 1000 °C; (**c**) 1100 °C; (**d**) 1200 °C; (**e**) 1300 °C.

The OM microstructure of austenite grains of samples held at different austenitizing temperatures for 1 min were taken as examples, as shown in Figure 3, to show the austenite grain boundaries. The grain sizes in all samples were relatively homogeneous, indicating that the growth of austenite grains is normal. The grain size of austenite with clear boundary was determined by the diagonal method. For the high accuracy of statistical data, a number of OM images were used to count austenite grains, among which at least 5 images were counted in Figure 3a–c, and at least 10 images were counted in Figure 3d,e. The average grain sizes of all samples are listed in Table 2. It is seen that the austenite grains grew little at 950 °C and 1000 °C even if prolonging the holding time to 120 min. Moreover, as observed from Figure 3c, austenite grains grew slightly when the austenitizing temperature was increased to 1100 °C. However, austenite grains were obviously coarsened with increasing the temperature to 1200 °C, as shown in Figure 3d.

In addition, it is also seen from Table 2 that the austenite grain size increased with increasing the heating temperature, and the coarsening of austenite grains was obvious when the temperature was above 1100 °C. The coarsening temperature of austenite grains is lower than the complete solution temperatures of TiC and TiN. During the heating process, precipitates dissolve after a certain temperature, leading to the decrease of pinning force of particles. Austenite begins to grow when the pinning force is smaller than the driving force although the particles are not completely dissolved at this time. Thus, the complete solution temperatures of TiC and TiN are generally higher than the grain coarsening temperature during the growth of austenite grains. Moreover, the austenite grain size increased with the extension of isothermal time at the austenitizing temperature, while it changed little when the isothermal time was more than one hour. The grain size of austenite was usually described with a log-normal distribution, as shown by Formula (18).

(18) $$\nu \left(d\right)=\frac{1}{\sqrt{2\pi }sd}exp(-\frac{{\left[\ln\left(d/d_{g}\right)\right]}^{2}}{2s^{2}})$$

where, *s* is the logarithmic standard deviation of grain size, *d* is the grain diameter, and *d_g_* is the median grain diameter.

**Figure 4:**
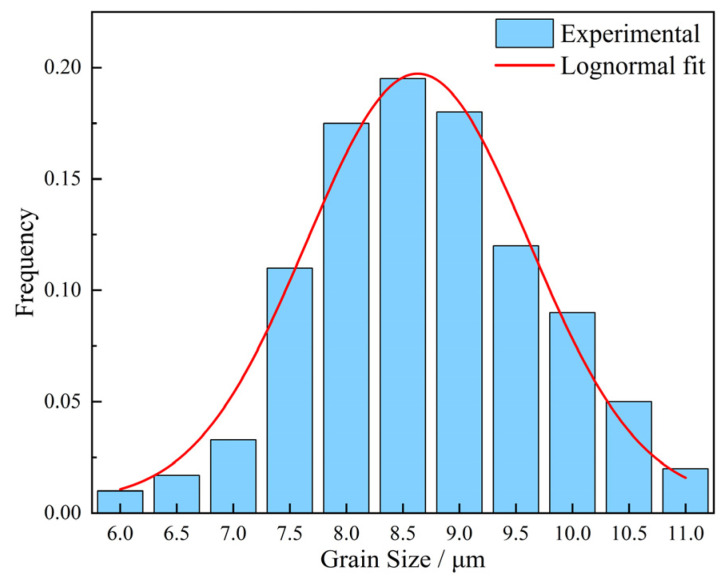
The distribution of austenite grain size of samples held for 1 min at the austenitizing temperature of 950 °C with a heating rate of 1 °C/s; the solid line indicates fitted log-normal distribution.

**Figure 5:**
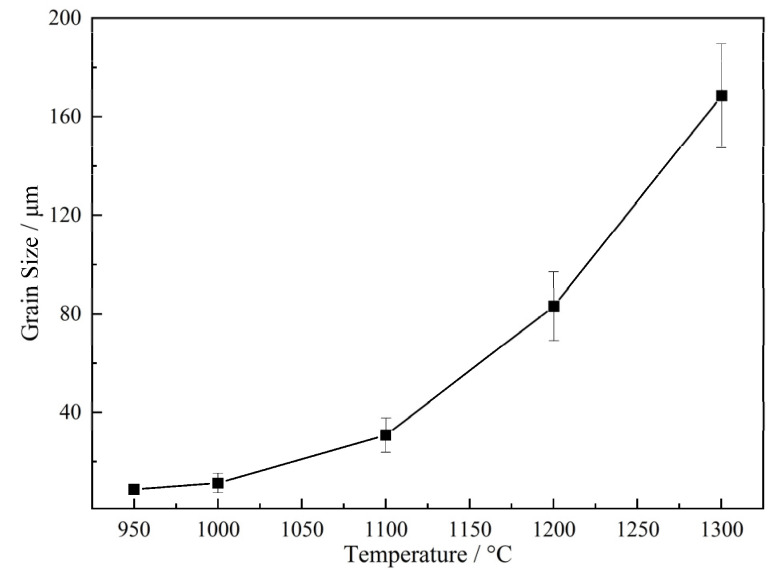
The change of grain size of original austenite with varied heating temperatures after holding for 1 min.

Figure 4 presents the austenite grain size distributions of samples held for 1 min at 950 °C with the heating rate of 1 °C/s. It indicates that the mean grain size and the width of the grain size distribution conform to log-normal distribution, independent of heating conditions. Figure 5 shows the change of grain size of austenite with different heating temperatures of samples holding for 1 min.

### 4.3. Precipitation Analysis

**Figure 6:**
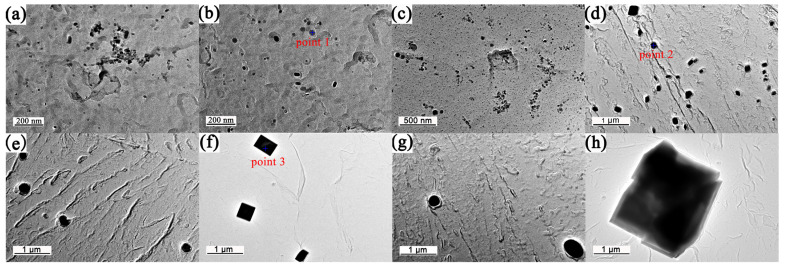
Precipitation analysis of different samples: (**a**) 950 °C–1 min; (**b**) 950 °C–120 min; (**c**) 1100 °C–1 min; (**d**) 1100 °C–120 min; (**e**) 1200 °C–1 min; (**f**) 1200 °C–120 min; (**g**) 1300 °C–1 min; (**h**) 1300 °C–120 min.

**Figure 7:**
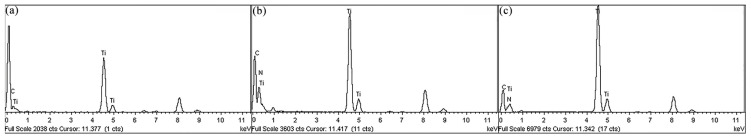
Precipitates energy spectrum analysis (**a**) spherical particle at point 1 in Figure 6b; (**b**) ellipsoidal particle at point 2 in Figure 6d; (**c**) cubic particle at point 3 in Figure 6f.

Figure 6 shows TEM images of precipitates in different samples. There were a variety of Ti containing precipitates with sizes ranging from 2 nm to 2 μm. By comparing Figure 6a with Figure 6b, it is found that with the increase of heating time, the precipitated particles decrease and distribute more evenly, which proves that the precipitates have not reached the equilibrium state at 950 °C for 1 min, and there is a continuous dissolving tendency of precipitates. Figure 6b shows that the precipitates still maintained round morphology after austenitization at 950 °C for 120 min. Moreover, the Ostwald ripening process was observed during a long time holding at 1100 °C, as presented in Figure 6c,d, in which the fine precipitates were redissolved, while the size of larger precipitates increased. As shown in Figure 6e,f, the precipitate size increased significantly and the precipitate morphology completely changed from round to square after holding at 1200 °C for a long time. It is reported that the growth behavior of the second phases, which were evenly distributed in the matrix and had strong chemical stability, mainly depended on the diffusion process of the controlled solute atoms [27]. Titanium carbides or nitrides have the same crystal structure of sodium chloride type with little difference in lattice constant. Therefore, titanium carbonitrides are usually miscible to form. Due to the existence of carbon film in carbon replica extraction samples, carbon peaks appeared inevitably in the energy spectrum. It is seen from the energy spectrum results (Figure 7) that the proportion of N element in the square precipitated particles increased. The solid solubility product of TiC was much higher than that of TiN (about 4–5 orders of magnitude) [28], thus TiC precipitates basically redissolved during the long-time heating process, while TiN precipitates still remained. The square particles only existed in the samples held at a high temperature for a long holding time (Figure 6d,f,h). Therefore, the composition of round particles was mainly TiC, and the composition of square particles was mainly TiN, as proved by the energy spectrums in Figure 7.

**Figure 8:**
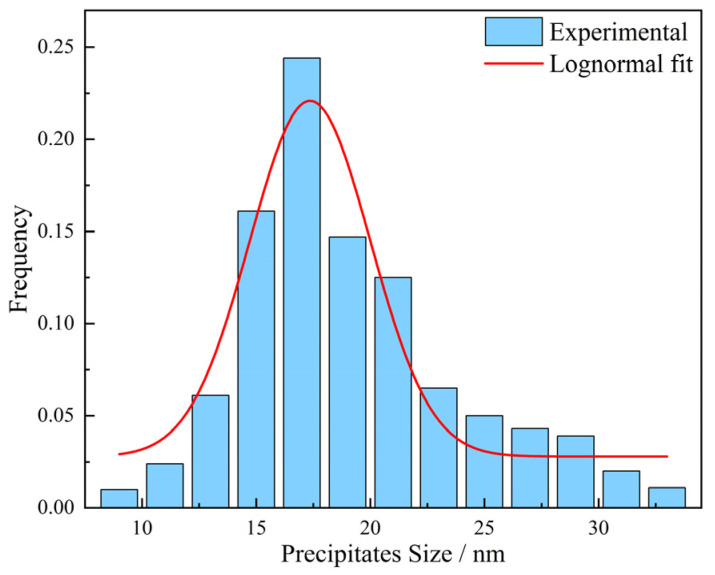
Precipitate size distribution for austenitizing temperature of 950 °C and a holding time for 1 min; the solid line indicates fitted log-normal distribution.

The size of precipitates in round shape was typically in the range of 2 to 200 nm. They were randomly distributed within grains. The size of elliptic precipitates was observed to be in the range of 80 to 300 nm. The square precipitates considered to be TiN have a size range of 200 nm~2 μm. The average precipitation sizes of samples with different austenitizing temperatures and times are shown in Table 3. Taking the sample holding at 950 °C for 1 min as an example, the measured precipitation size distribution can be well represented by a log-normal distribution, as illustrated in Figure 8.

(19) $$\nu \left(r\right)=\frac{1}{\sqrt{2\pi }sr}exp(-\frac{{\left[\ln\left(r/\mu \right)\right]}^{2}}{2s^{2}})$$

where *ν(r)* is the probability density of a log-normal particle size distribution, *r* is the individual particle radius, *μ* is the median radius of the distribution, and *s* is the standard deviation.

## 5. Verification of Model and Discussion

**Figure 9:**
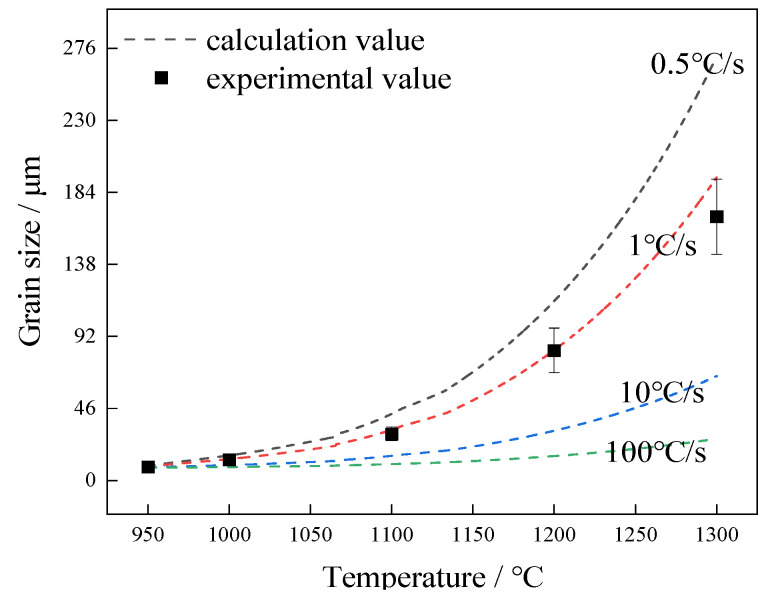
The comparison between the calculated and experimental values. Dashed line shows the calculation results, and solid square symbol shows the experimental results.

The austenite grain size at different heating temperatures with the heating rate of 1 °C/s was calculated as shown in Figure 5. The results are presented in Figure 9 by solid square symbol. The dashed lines calculated by the method described in Section 2 in Figure 9 show the variation curves of austenite grain size with varied heating temperatures at different heating rates of 0.5, 1, 10, 100 °C/s.

The calculation process is as follows. Firstly, the minimum volume fraction ($f_{v-min}$) of the particles in austenite reaching the precipitation-dissolution equilibrium (which varies with the temperature) is obtained by calculating the solid solubility product formula with the composition of the steel (Equations (3) and (4)). Secondly, $f_{v-min}$ is set as the boundary condition for the precipitation-dissolution equilibrium. Then, the volume fraction of the precipitates in the dissolution state (i.e., non-equilibrium state) is calculated by Formula (8). If $f_{v}$ is larger than the $f_{v-min}$, it indicates that the particles have not reached the precipitation-dissolution equilibrium state at this time. Otherwise, the precipitation solution reaches an equilibrium state at this time, and the $f_{v}$ of precipitation is the minimum value and does not change with the extension of time at the fixed temperature. Next, the average size *r* of the precipitates is calculated by Formula (9). Finally, the austenite grain size *D* is calculated by Formula (17). It should be pointed out that the temperature is a function of time and related to the heating process. The overall calculation process is completed by designing the time node of 1 s through Matlab R2024a software.

When the holding time is only 1 min, the dissolution of the second phase precipitates has not reached the equilibrium state, and the austenite grain size has not reached the maximum value at the temperature. Therefore, the austenite grain size is mainly controlled by Ti diffusion. The faster the heating rate, the smaller the austenite grain size at the same isothermal temperature (Figure 9). With the increase of heating rate, the time for reaching the target temperature decreases, thus the diffusion time of precipitated elements (*t* in Formula (5)) decreases and the maximum distance (*L_max_*) between precipitated particles decreases. The short diffusion time results in less dissolution of precipitates. Therefore, the strong pinning effect of precipitates on austenite grains and the great hindrance to austenite grain boundary migration still remain. Meanwhile, the migration time for austenite grain boundary is short, which leads to the insufficient migration of austenite grain boundary. Above factors result in smaller austenite grains. On the other hand, when the heating speed is 0.5 °C/s or even slower, the sample is almost in equilibrium state during the heating process, resulting in larger austenite grain size. Comparing the experimental data with the calculated values, the proposed model has good prediction results.

It is observed in Figure 9 that when the heating temperature exceeds 1150 °C, the austenitic grains of the experimental steel are obviously coarsened, which corresponds to the results of the metallographic structure in Figure 2. The results in reference [29] have shown that austenite coarsening of low carbon microalloyed steels is mainly related to the dissolution and coarsening of the second phase particles. For the experimental steel in the present study, the starting temperature of austenite coarsening is between 1100 °C and 1200 °C, and both experimental and calculation results show that the precipitation and dissolution mainly occur in this temperature range.

Most previous studies focused on predicting the grain size of austenite in equilibrium state at a certain temperature [30,31,32], while the calculation method of austenite grain growth proposed in the present study can predict the grain size of austenite during the heating process well, providing a new idea for the prediction of austenite grain size during the heating process.

## 6. Conclusions

In this study, the solid solubility product formula, the Ti element diffusion principle and the Ostwald ripening formula were combined to generate a precipitation model, which was used to analyze the dissolution and precipitation behaviors of precipitates in Ti-microalloyed steels. Then, by combining the precipitation model with the austenite growth model, a model predicting the austenite grain size during the heating process was proposed. The results of continuous heating experiments were used to verify the accuracy of the proposed model. The main conclusions are as follows:

(1) A prediction model of the austenite grain growth in Ti-containing microalloyed steels at different heating rates was established, providing a new idea for the prediction of austenite grain size in non-equilibrium state during the heating process. The traditional austenite growth models are only applicable to the corresponding experimental steels. Compared with the traditional austenite grain growth model, the proposed model has a wide range of applications, and can be directly used to estimate the austenite grain size of Ti-containing microalloyed steels during the heating process.

(2) The austenite grain size of Ti-containing microalloying steels increases with increasing the heating temperature, while it remains unchanged with further increasing isothermal time after certain holding time, which is related to the balance of the driving force and the pinning force of grain boundary migration. The austenite dramatic coarsening temperature of the experimental steel is between 1100 °C and 1200 °C.

## Figures and Tables

**Table 1:** The values of *k* and *n* in Formula (16) in different literatures.

Literature	*k*	*n*
Zener-Smith [22]	4/3	1
Gladman [24]	π/6(1.5–2/Z), $\sqrt{2}$ ≤ Z ≤ 2	1
Srolovitz-Anderson [25]	4/9	1/2
Nishizawa [23]	4/3, 8/3	2/3

**Table 2:** Statistical results of average grain size of austenite in all quenched samples.

T(°C)	1 min		15 min		30 min		60 min		120 min	
	Grain Size(μm)	Standard Deviation	Grain Size (μm)	Standard Deviation	Grain Size (μm)	Standard Deviation	Grain Size (μm)	Standard Deviation	Grain Size (μm)	Standard Deviation
950	8.8	0.52	9.8	0.53	11.2	0.52	12.1	0.47	13.0	0.49
1000	11.3	0.55	12.1	0.51	13.5	0.42	15.9	0.49	16.1	0.50
1100	30.8	0.56	33.5	0.47	39.5	0.43	48.6	0.44	49.8	0.50
1200	86.1	0.48	104.1	0.43	123.6	0.54	141.9	0.55	142.4	0.59
1300	168.5	0.60	171.5	0.57	205.6	0.57	233.5	0.59	234.1	0.62

**Table 3:** Statistical results of average precipitate size.

T (°C)	1 min		120 min	
	Precipitate Size (nm)	Standard Deviation	Precipitate Size (nm)	Standard Deviation
950	18.2	0.42	38.3	0.61
1000	23.3	0.56	84.5	0.59
1100	34.8	0.59	198	0.55
1200	268	0.51	479	0.54
1300	1145	0.60	1862	0.56

## Data Availability

The original contributions presented in the study are included in the article, further inquiries can be directed to the corresponding authors.
